# Measuring the Intensity of Stress Experienced and Its Impact on Life in Patients with Diagnosed Alcohol Use Disorder

**DOI:** 10.3390/jcm13020572

**Published:** 2024-01-19

**Authors:** Mateusz Curyło, Aleksandra Czerw, Marlena Rynkiewicz-Andryśkiewicz, Przemysław Andryśkiewicz, Marcin Mikos, Olga Partyka, Monika Pajewska, Jakub Świtalski, Katarzyna Sygit, Marian Sygit, Beata Karakiewicz, Elżbieta Cipora, Mateusz Kaczmarski, Mariola Głowacka, Łukasz Strzępek, Jarosław Drobnik, Piotr Pobrotyn, Edyta Krzych-Fałta, Ewa Bandurska, Weronika Ciećko, Anna Knyszyńska, Sławomir Porada, Monika Borzuchowska, Remigiusz Kozlowski, Michał Marczak

**Affiliations:** 1 Department of Internal Medicine, Rehabilitation and Physical Medicine, Medical University of Lodz, 90-647 Lodz, Poland; 2 Medical Rehabilitation Department, The Ministry of the Interior and Administration Hospital, 30-053 Cracow, Poland; 3 Department of Economic and System Analyses, National Institute of Public Health NIH—National Research Institute, 00-791 Warsaw, Poland; 4 Department of Health Economics and Medical Law, Medical University of Warsaw, 02-091 Warsaw, Poland; 5 Department of Treatment of Alcohol Abstinence Syndromes, Independent Public Healthcare Facility in Lezajsk, 37-300 Lezajsk, Poland; 6 Faculty of Medicine and Health Sciences, Andrzej Frycz Modrzewski Cracow University, 30-705 Cracow, Poland; 7 Faculty of Health Sciences, Calisia University, 62-800 Kalisz, Poland; 8 Subdepartment of Social Medicine and Public Health, Department of Social Medicine, Faculty of Health Sciences, Pomeranian Medical University in Szczecin, 71-210 Szczecin, Poland; 9 Medical Institute, Jan Grodek State University in Sanok, 38-500 Sanok, Poland; 10 Collegium Medicum, The Mazovian Academy in Płock, 09-402 Płock, Poland; m.glowacka@mazowiecka.edu.pl; 11 Clinical Department of General and Oncological Surgery, Saint Raphael Hospital, 30-693 Cracow, Poland; 12 Department of Family Medicine, Faculty of Medicine, Wroclaw Medical University, 51-141 Wroclaw, Poland; 13 Remedial Specialistic Clinic “Pulsantis Sp z o.o”, 53-238 Wroclaw, Poland; 14 Department of Basic of Nursing, Faculty of Health Sciences, Medical University of Warsaw, 01-445 Warsaw, Poland; 15 Center for Competence Development, Integrated Care and e-Health, Medical University of Gdansk, 80-204 Gdansk, Poland; 16 Department of Functional Diagnostics and Physical Medicine, Pomeranian Medical University in Szczecin, 71-210 Szczecin, Poland; 17 Institute of Health Sciences, Medical College of Rzeszow University, 35-315 Rzeszow, Poland; 18 Department of Management and Logistics in Healthcare, Medical University of Lodz, 90-131 Lodz, Poland; 19 Collegium of Management, WSB University in Warsaw, 03-204 Warsaw, Poland

**Keywords:** addiction, alcohol, stress, sense of coherence

## Abstract

Alcohol addiction is characterized by extensive alcohol consumption that dominates other behaviours previously important to a patient. According to data from The State Agency for Prevention of Alcohol-Related Problems, up to 900,000 people in Poland are addicted to alcohol. On average, approximately 9.7 L of pure alcohol per capita was consumed in 2021. Alcohol addiction may cause severe health problems and is one the key risk factors for various diseases. Stress plays an important role in the process of alcohol addiction and is also a predictor for lower enjoyment in life. On the other hand, sense of coherence may be a stronger protective factor. The aim of our study was to verify the relation between the level of perceived stress among patients with alcohol addiction and satisfaction with life. Because sense of coherence is a disposition that allows for managing stress effectively, the latter should be reflected in the results of multivariate analyses that take both the level of stress and sense of coherence into account. In the present study, sense of coherence and perceived stress were negatively correlated; therefore, strengthening internal resources for managing difficult and stressful situations is recommended.

## 1. Introduction

Alcohol addiction can be defined as a set of physiological, behavioural, and cognitive phenomena in which alcohol drinking dominates other behaviours. The prevalence rate of alcohol dependence in Poland is estimated to be 2.2%, with 95% confidence intervals falling in the range of 1.8–2.7% [1]. When using routine hospital discharge data for the ICD-10 diagnostic categories F10 and K70, the estimate is even higher and equal to 4.8, with 95% CI (3.9–5.7%) [2]. At the same time, the number of litres of pure alcohol consumed per person aged 15+ per year in Poland gradually increases [3]. It was equal to 9.2 L in 2000, 10.6 L in 2005, 11.2 L in 2010, and 11.6 L in 2018. When an exponential smoothing algorithm is applied to these data, the estimates are 12.5 L for 2023 and 13.1 L for 2028. There is a trend of increasing consumption of alcoholic products sold in small packages—100 mL and 200 mL. According to research, such small quantities are bought by 3 million people in Poland every day. According to OECD statistics, in 2018, 2.4 bottles of wine or 4.5 L of beer per person over 15 years of age was consumed in Poland per week [3]. The effects of alcohol consumption result in direct costs related to health care, indirect costs related to absenteeism from work, and social costs resulting from changes in social arrangements [4]. People with heavy drinking episodes show greater absenteeism from work. In addition, alcohol increases the phenomenon of presenteeism and unsafe behaviour at work. Daily alcohol consumption is also associated with dysfunctions in the area of social life, such as the breakdown of interpersonal relationships [5]. The total social costs of alcohol consumption estimated on the basis of a percentage of GDP could reach PLN 45 billion [6].

There was a global increase in alcohol consumption during the COVID-19 pandemic. It had a wide impact on many aspects of people’s economic and social life, creating burdens that could increase the intensity of risky behaviour in some people. Research regarding the pandemic period indicated two possible scenarios for alcohol consumption: a decrease in alcohol consumption resulting from limited availability or an increased alcohol consumption due to mental anxiety. Some studies suggested a negative impact of COVID-19 on the development of anxiety or mood disorders [7]. Mental health disorders had the greatest potential impact on the increased use of psychoactive substances, including alcohol. The greatest period of increased alcohol consumption occurred during the strictest sanitary and epidemiological restrictions [8]. The COVID-19 pandemic may have con-tributed to the creation of an environment where stress, other strong emotions and being in negative situations (domestic violence, loss of employment) may have distorted the perception of alcohol consumption and led to its abuse [9]. Studies also indicated that the pandemic increased the risk of alcohol use disorder recurrence in patients [10].

Alcohol abuse has various negative consequences on one’s health, including injuries, brain damage, or liver disease and also has a negative impact on psychological functioning and social behaviours [11]. The World Health Organization estimates that alcohol use disorder is the third cause of disability in highly developed countries [12]. It is also associated with lower life satisfaction [13,14,15]. Substance use, including drinking alcohol, can conceptualize as a stress coping strategy since alcohol consumption may demonstrate anti-anxiety properties. [16]. Therefore, in a group of alcohol-dependent patients, one can expect the level of perceived stress to be positively associated with alcohol craving and severity of dependence and, at the same time, negatively related to life satisfaction [17]. 

Antonovsky developed the concept of sense of coherence to enhance understanding effective management of a high level of stress without severe consequences for one’s well-being [18]. The concept refers to perception of one’s situation as understandable, manageable, and meaningful. As a consequence, stressful situations are experienced as challenges that are worth commitment and dedication. A related concept of personal growth which can be understood as sense of coherence in a personal context was already shown to be positively related to satisfaction with life in a group of adults diagnosed with alcoholism [19]. Ego-resiliency and optimal regulation were also positively related to satisfaction with life in a study involving individuals with alcohol dependence [13]. It was also shown that sense of coherence was positively related to health behaviours in a group of men with alcohol addiction; however, this effect was moderated by participants’ age [20].

The present study was designed to confirm that the level of perceived stress in a group of alcohol-dependent patients is negatively related to satisfaction with life and to verify if sense of coherence is a stronger predictor of life satisfaction than level of stress. Because sense of coherence is a disposition that allows for managing stress effectively, the latter should be reflected in the results of multivariate analyses that take both level of stress and sense of coherence into account. Lastly, because the age of the alcohol-dependent patients was detected as a moderator of associations involving sense of coherence, the moderation effect of age was also verified in this study.

## 2. Materials and Methods

### 2.1. Participants

The sample consisted of 104 participants aged 25–70 (M = 42.78; SD = 10.57): 89 males aged 26–70 (M = 42.77; SD = 10.44) and 13 females aged 25–63 (M = 42.85; SD = 11.80). Two participants did not provide the information about their gender. All participants were patients at the Department of Treatment of Alcohol Abstinence Syndromes in Lezajsk, Poland. The study took place in August–September 2022.

The participants completed three questionnaires: PSS-10, SOC-29, and SWLS. The PSS-10 (Perceived Stress Scale) questionnaire was used for measuring the intensity of experienced stress [21,22]. The questionnaire consists of 10 questions, each scored from 0 to 4. The sum of scores for the answers gives a total score within the range of 0–40 points. The higher the score, the higher the level of experienced stress. The reliability of the measurement with the use of PSS-10 in terms of Cronbach’s α coefficient in this study was equal to 0.87.

SOC-29 was used to measure sense of coherence [18]. The questionnaire consists of 29 questions, each scored from 1 to 7. The sum of scores for the answers gives a total score within the range of 29–203 points. The higher the score, the higher the sense of coherence. However, sense of coherence is considered to be a multidimensional construct. Therefore, in addition to the total score, the questionnaire also allows for calculating scores on three subscales, namely, comprehensibility (the cognitive aspect of sense of coherence), manageability (the instrumental dimension), and meaningfulness (the motivational dimension). The scores for the three subscales are based on 11 items, 10 items, and 8 items, respectively. The reliability of the measurement with the use of SOC-29 in the present study was α = 0.78 for comprehensibility, α = 0.75 for manageability, α = 0.79 for meaningfulness, and α = 0.89 for general sense of coherence.

The SWLS (Satisfaction with Life Scale) questionnaire was used for measuring life satisfaction [22]. The questionnaire consists of 5 questions, each scored from 1 to 7. The sum of scores for the answers gives a total score within the range of 5–35 points. The higher the score, the higher the level of life satisfaction. The reliability of the measurement with the use of SWLS in the present study was equal to α = 0.81.

### 2.2. Statistical Analysis

In the first step, descriptive statistics, including measures of skewness and kurtosis, were used to assess possible deviations from the normal distribution. The level of perceived stress and satisfaction with life acquired in the present study was then compared with other studies. In the next step, a correlation analysis was performed. The level of perceived stress and sense of coherence were analysed as predictors of satisfaction with life. This analysis was based on a regression analysis performed with the use of a stepwise method and a hierarchical model using the enter method. Finally, participants’ age was analysed as a moderator of relationships between perceived stress, sense of coherence, and satisfaction with life. A moderation analysis was performed using Hayes’ macro Process model no. 1 (2018).

## 3. Results

### 3.1. Descriptive Statistics

Table 1 presents descriptive statistics for analysed variables, i.e., mean values, standard deviations, minimum and maximum values, measures of skewness, and kurtosis.

The values for measures of skewness and kurtosis did not indicate substantial deviation from the normal distribution. Therefore, subsequent analyses were based on parametric statistical methods. 

### 3.2. Correlation Analysis

Table 2 presents the values of Pearson’s correlation coefficients between analysed variables, including participants’ age.

The level of perceived stress correlated negatively with satisfaction with life. All dimensions of sense of coherence correlated positively with satisfaction with life. Participants’ age did not correlate with the other variables.

Perceived stress, sense of coherence, and participants’ age were analysed as predictors of satisfaction with life. Many statistically significant correlations between analysed variables were detected (see Table 2). Therefore, to avoid multicollinearity, the stepwise algorithm of regression analysis was applied. The results are presented in Table 3 below.

General sense of coherence was the only predictor of satisfaction with life. The relationship was positive. It explained 16.8% of satisfaction with life variance.

To understand the relationship between the analysed variables better, a hierarchical regression model was implemented. The level of perceived stress was entered in the first block and then sense of coherence was added in the second block. The results are depicted in Table 4.

The negative relationship between the level of perceived stress and satisfaction with life was not statistically significant when sense of coherence was added to the model in the second block. The value of the standardized regression coefficient for perceived stress dropped from −0.34 to −0.17. Sense of coherence and perceived stress were negatively correlated; however, the value of variance inflation factor (VIF) acquired in the second block of the regression model was equal to 1.58, indicating no significant multicollinearity. When the level of perceived stress was controlled, sense of coherence explained 5.3% of satisfaction with life variance.

The age of participants did not correlate with any other analysed variables. In the next step, the age of participants was analysed as a moderator of relationships between the dimensions of sense of coherence and satisfaction with life. Table 5 presents the values acquired for the interaction effects between participants’ age, perceived level of stress, and each of sense of coherence dimension from the moderation analysis. 

None of the interaction effects were statistically significant, which meant that the positive relationship between sense of coherence and satisfaction with life, but also the negative relationship between the level of perceived stress and satisfaction with life, did not depend on participants’ age.

## 4. Discussion

The mean value of perceived stress among the 89 males in this study measured with the use of PSS-10 was 21.79 (SD = 6.92). In a study by Bejda et al., the level of perceived stress was measured also with the use of PSS-10 in a group of 170 males addicted to alcohol (aged 60–68) [23]. The mean value acquired in the study was 23.5 (SD = 3.7). According to the value of the independent samples *t*-test, the difference between the present study and the results acquired by Bejda et al. was statistically significant (t(257) = 2.59, *p* < 0.05, and d = 0.31), meaning that the level of perceived stress in the present study was significantly lower than that observed in the Bejda et al. study. However, the effect size was rather small.

Regarding satisfaction with life, the mean value among the 89 males in the present study measured with the use of the SWLS was 18.02 (SD = 5.70). Bejda et al. also measured satisfaction with life using SWLS in a group of 170 males addicted to alcohol (aged 60–68). The mean value acquired in the study was 17.2 (SD = 4.9). According to the value of the independent samples t-test, the difference between the present study and the results acquired by Bejda et al. was not statistically significant (t(257) = 1.10, *p* > 0.05, d = 0.15), meaning that the level of satisfaction with life in the present study was close to the level acquired in the Bejda et al. study [23]. 

In a study conducted by Dębski, satisfaction with life was measured with the use of the SWLS in a group of 60 alcohol-dependent patients and in a group of 40 healthy controls [13]. The mean values acquired were 15.00 (SD = 5.72) in the group of alcohol-dependent patients and 21.50 (SD = 6.53) in the group of healthy controls. According to the values of the independent samples t-test, the mean value for satisfaction with life (17.96, SD = 6.19) was significantly higher than that of the group of alcohol-dependent patients (t(162) = 3.03, *p* < 0.01, d = 0.50) and significantly lower than that of the group of healthy controls (t(142) = 3.03, *p* < 0.01, d = 0.56) in Dębski’s study. In both cases, the differences were moderate effects [13].

The relationship between stress levels and alcohol consumption has been investigated since the beginning of alcohol addiction research [24,25]. Stress is considered as a trigger for relapse and a significant factor that promotes increased motivation to drink [26]. The COVID-19 pandemic has resulted in an overall increased sense of stress in society and reduced life satisfaction. The comparisons regarding the level of perceived stress and satisfaction with life revealed that the level of stress in the current sample was close to the level acquired in other studies involving alcohol-dependent patients [7,26,27]. However, the level of life satisfaction can be higher than that observed in other studies. Still, it was lower than the level characteristic for healthy controls. It was confirmed, as expected, that the level of perceived stress was negatively related to life satisfaction. Sense of coherence was related to life satisfaction positively. This applied to all coherence dimensions; however, the relationship involving general sense of coherence was the strongest. The results acquired showed that general sense of coherence is a better predictor of satisfaction with life than the level of perceived stress. In fact, when general sense of coherence was taken into account, the level of perceived stress was no longer related significantly to life satisfaction. This finding corresponds well with the results of other studies concerned with analysing the significance of psychological concepts that are close to sense of coherence for life satisfaction of alcohol-dependent patients [28,29,30,31]. This applies to concepts like personal growth, ego-resiliency, and optimal regulation [13,19,29]. No significant effects of participants’ age were detected, which means that as far as the results of the present study are concerned, there is no indication of age restricting the generalization of the potential impact of sense of coherence for life satisfaction, contrary to what was found regarding the health behaviours [32].

## 5. Limitations

There are some limitations regarding the conclusions drawn from the present study. Firstly, the study was cross-sectional. Sense of coherence is not a concept involving significant changes in time, but the level of perceived stress is. The longitudinal study could verify if alcohol-dependent patients who are more coherent are also more stable regarding their satisfaction with life when the level of perceived stress changes. A study conducted on a larger sample could be more conclusive regarding the effect of participants’ age. All effects of age in the present study were statistically insignificant; however, Pearson’s correlation coefficient between age and satisfaction with life (0.191, see Table 2) would be statistically significant in a study conducted on a sample of 212 participants. A correlation of this magnitude means that only 3.6% of variance is explained. This could still enhance the precision of estimates acquired in a model for predictors of life satisfaction.

During this study, no information was collected on the patients’ pharmacological treatment, which may influence the respondents’ answers. For this reason, we are unable to take into account the role of administered medications on the sense of declared life satisfaction, the level of stress felt, or the level of sense of coherence.

## 6. Conclusions

The link between stress and alcohol consumption is complex and involves a number of factors that influence the outcome of stress and alcohol interaction. In the present study, sense of coherence and perceived stress were negatively correlated; therefore, strengthening internal resources for managing difficult and stressful situations is recommended, especially among patients undergoing addiction treatment. General sense of coherence could be a predictor of satisfaction with life. However, more complex studies, especially a longitudinal study with a larger group of patients, is required to further prove the correlation between those variables among patients with alcohol addiction.

## Figures and Tables

**Table 1:** Descriptive statistics for analysed interval variables.

Variables	M	SD	Min	Max	S	K
Perceived stress	21.79	6.92	4	38	−0.24	−0.29
Comprehensibility	42.20	9.97	16	63	−0.27	−0.44
Manageability	45.39	9.06	26	65	−0.05	−0.40
Meaningfulness	39.90	8.42	13	56	−0.69	0.41
Sense of coherence	127.50	23.13	63	176	−0.25	−0.30
Satisfaction with life	17.96	6.19	3	31	0.15	−0.52

M—mean value; SD—standard deviation; min—minimum value; max—maximum value; S—measure of skewness; K—measure of kurtosis.

**Table 2:** Correlation coefficients between analysed variables.

Variables	1.	2.	3.	4.	5.	6.
1. Perceived stress	-	-	-	-	-	-
2. Comprehensibility	−0.576 **	-	-	-	-	-
3. Manageability	−0.548 **	0.666 **	-	-	-	-
4. Meaningfulness	−0.419 **	0.485 **	0.528 **	-	-	-
5. Sense of coherence	−0.616 **	0.869 **	0.871 **	0.780 **	-	-
6. Satisfaction with life	−0.342 **	0.314 **	0.321 **	0.353 **	0.390 **	-
7. Age	−0.102	0.024	0.077	0.031	0.051	0.191

** *p* < 0.01.

**Table 3:** Analysis of key predictors of satisfaction with life.

Dependent Variable	Predictors	B	t	*p*	ΔR^2^
Satisfaction with life	Sense of coherence	0.41	4.36	0.001	0.17

B—standardized regression coefficient; t—value of test for predictor’s significance; *p*—statistical significance; ΔR^2^—change in determination coefficient when the predictor added to a model.

**Table 4:** Analysis of perceived stress and sense of coherence as predictors of satisfaction with life.

Block	Dependent Variable	Predictors	B	t	*p*	ΔR^2^
Block 1	Satisfaction with life	Perceived stress	−0.34	−3.58	0.001	0.12
Block 2	Satisfaction with life	Perceived stress	−0.17	−1.42	0.158	
		Sense of coherence	0.29	2.48	0.015	0.05

B—standardized regression coefficient; t—value of test for predictor’s significance; *p*—statistical significance; ΔR^2^—change in determination coefficient when the predictor was added to a model.

**Table 5:** Interaction effects between participants’ age, perceived level of stress, and each of sense of coherence dimension.

Interaction Effects	B	t	*p*
Perceived stress × Age	0.16	1.58	0.118
Comprehensibility × Age	−0.13	−1.20	0.233
Manageability × Age	−0.07	−0.69	0.493
Meaningfulness × Age	−0.03	−0.30	0.768
Sense of coherence × Age	−0.10	−0.98	0.329

B—standardized regression coefficient; t—test for statistical significance of regression coefficient; *p*—statistical significance.

## Data Availability

The original data collected to conduct this study could be made available from the authors.
